# Supplementary material for: Further Delineation of Phenotype and Genotype of Primary Microcephaly Syndrome with Cortical Malformations Associated with Mutations in the WDR62 Gene
Source: Genes (Basel). 2021 Apr 19;12(4):594. doi: 10.3390/genes12040594 (PMC8072659; doi:10.3390/genes12040594)
Supplement: Supplementary file 1 [file genes-12-00594-s001.zip › genes-1145756-supplementary.pdf]

Supplementary Materials

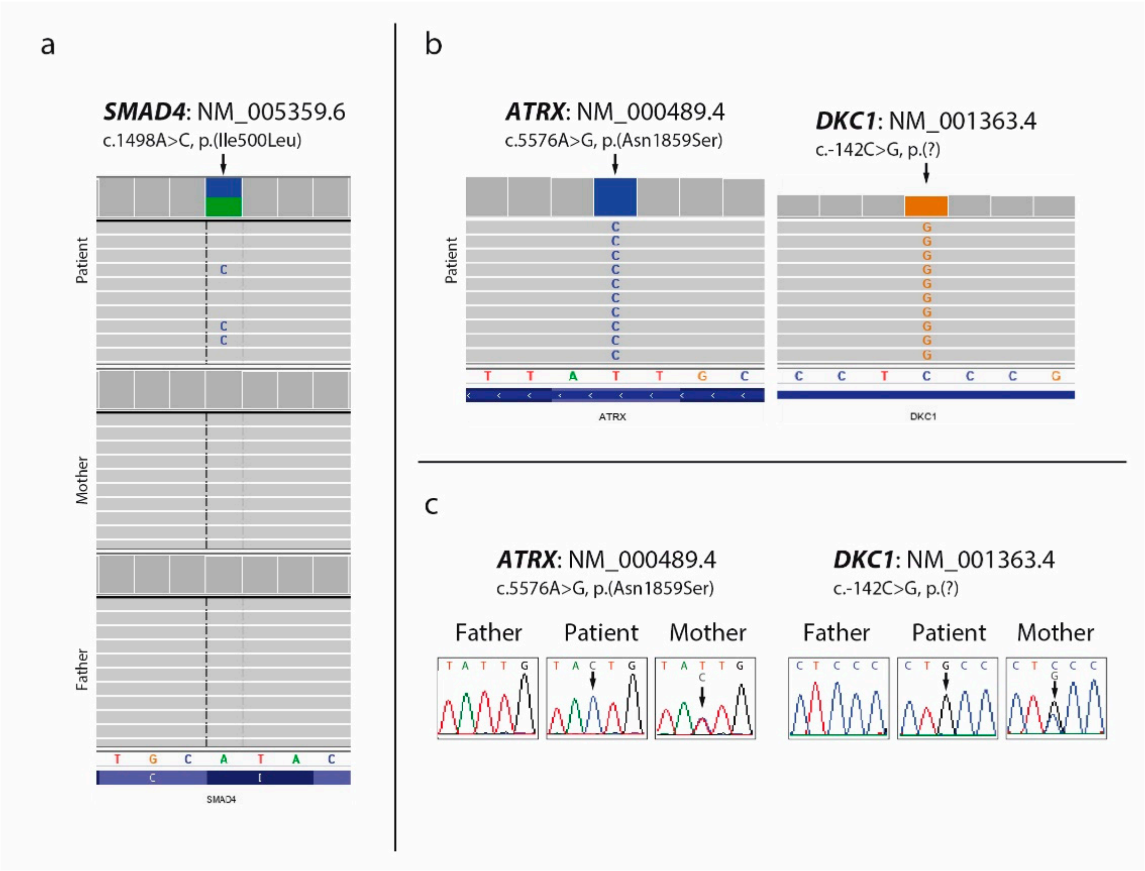

Figure S1. The Integrative Genomics Viewer (IGV) from Amplicon Deep Sequencing (**a**), IGV from Whole Exome Sequencing (**b**) an Sanger confirmation (c) of additional WES molecular findings. IGV of c.1498A>C, p.(Ile500Leu) variant found in *SMAD4* gene of case 2 (**a**). IGV and Sanger sequencing plots of p.5576A>G, p.(ASN1859Ser) variant found in *ATRX* gene of case 3, and c.-142C>G (p.?) variant found in *DKC1* gene of case 3 (**b**,**c**).

Table S1. Summary of the published mutations in the *WDR62* gene.

No	Mutation (DNA level)	Mutation (amino acid level)	Exon/Intron	Homo-/heterozygoticty	Type mutation	Population	References
1	c.28G>T	p.Ala10Ser	1	ht	M	chinese	7
2	c.189G>T	p.Glu63Asp	2	ht	M	chinese	7
3	c.193G>A	p.Val65Met	2	h	M	saudi	22,29
4	c.194T>A	p.Val65Glu	2	ht	M	polish	case 2
5	c.332G>C	p.Arg111Thr	3	h	M	pakistani	13
6	c.363delT	p.Asp112Metfs*5	4	h	fs	mexican	29
7	c.390G>A	p.Glu130Glu	4	h	M	sudan	8
8	c.535_536insA	p.Met179fs*21	5	h	fs	indian	9
9	c.617G>C	p.Trp224Ser	6	h	M	turkish	10
10	c.668T>C	p. Phe223Ser	6	h	M	romani	27
11	c.731C>T	p.Ser244Leu	7	ht	M	japanese	17
12	c.797C>T	p.Ala266Val	7	ht	M	saudi	20
13	c.900C>A	p.Cys300*	8	h	N	indian	9
14	c.883-1273_850del	del ex 8-9	8-9	h	del	pakistani /korean	25
15	c.883-4_890del	del intron 7 +exon8	8	ht	del	korean	24
16	c.1027C>T	p. Gln343*	8	h	N	morrocan	30
17	c.1043+1G>A	p.Ser348Argfs*63	8	h	ss	turkish	29
18	c.1102G>A	p.Asp368Asn	9	ht	M	saudi	20
19	c.1143delA	p.His381Profs*48	9	h	fs	pakistani	16
20	c.1194G>A	p.Trp398	9	h	M	pakistani	12,13
21	c.1198G>A	p.Glu400Lys	9	h	M	spanish	6
22	c.1313G>A	p.Arg438His	10	h	M	pakistani, german	11,12,13,14
23	c.1408C>T	p.Gln470*	11	h	N	turkish	10
24	c.1531G>A	p. Asp511Asn	11	h	M	pakistani	14,22
25	C1535G>A	p.Arg512Gln	11	ht	M	chinese	26
26	c.1576G>A	p.Glu526Lys	12	h	M	turkish	10
27	c.1576G>T	p.Glu526*	12	h	N	turkish	10
28	c.1605_1606insT	p. Glu536*	12	h	N	turkish	23
29	c.1642+2T>g	p.?	12	ht	ss	polish	Case 1
30	c.1684C>G	p.His562Asp	13	ht	fs	korean	24
31	c.1711_1712insTA	p.Asn571Ilefs*27	13	ht	N	polish	Case 3
32	c.1777_1778del GA	p.Asp593Hisfs*9	13	ht	N	polish	Case 1
33	c.1821dupT	p.Arg608Serfs*26	14	h	fs	french canadian	15
34	c.1942C>T	p.Gln648*	15	h	M	pakistani	14
35	c.2083delA	p.Ser696Alafs*4	17	ht	fs	?	18
36	c.2195C>T	p.Thr732Ile	20	h	M	pakistani	12
37	c.2413G>T	p.Glu805*	20	ht	N	japanese	17
38	c.2520+5G>T	p.Asp823Alafs*5	21	h	ss	pakistani	25
39	c.2527dup G	p.asp843glyfs*3	22	h	fs	pakistani	24
40	c. 2588G>A	p.Arg863His	22	h	M	tunisian	23
41	c.2618dupT	p.Lys874Glnfs*40	22	ht	fs	chinese, korean	26,28
42	c.2667_2668GA>TT	p.[Met8891Ile;Lys890*]	22	h		pakistani	25
43	c.2472_2473del AG	p.Gln918 Glyfs*18	23	ht	fs	north european	18
44	?	p. Asp955Alafs*111	23	h	fs	?	23
45	c.2864_2867delACAG	p.Asp955Alafs*112	23	ht	N	polish, german	Case 3, 11

46	c.2867+4_2867+7delGGTG	Ser956Cysfs*38	23 in	h	ss	turkish	29
47	c.3232G>A	p.Ala1078Thr	27	h	mis	pakistani	22
48	c.3335+1G>C	?	intr	h	ss	italian	19
49	c.3361delG	p.Ala1121Glnfs*6	28	h	fs	pakistani	12,13
50	c.3503G>A	p.Trp1168	29	h	M	pakistani	13
51	c.3839_3855delCAG…	p.Gly1280Alafs*21	30	h	fs	turkish / pakistani	10,29
52	c.3878C>A	p.Ala1293Asp	30	h	M	saudi	21
53	c.3936_3937insC	p.Val1314Glyfs*17	30	h	fs	turkish	14,29
54	c.3936dupC	p.Val1314Argfs*18	30	h	N	caucasian	12,22
55	c.4205delTGCC	p.Val1402Glyfs*12	31	h	N	turkish	10
56	c.4241dupT	p.Leu1414Leufs*41	31	h	fs	pakistani, german	12,22
57	c.4258C>T	p.Gln1420*	31	ht	N	polish	Case 2

h: homozygote, ht: heterozygote, N: nonsense, fs: frameshift, M: missense, del: deletion, ss: splice site mutation.
